# The effects of stocking density on slaughter performance and some meat quality parameters of Pekin ducks

**DOI:** 10.5194/aab-65-199-2022

**Published:** 2022-05-10

**Authors:** Sabri Arda Eratalar, Nezih Okur, Ahmet Yaman

**Affiliations:** Faculty of Agriculture, Department of Poultry Science, Golkoy Campus, Bolu Abant Izzet Baysal University, Bolu, Türkiye

## Abstract

The effects of stocking density on slaughter performance and meat quality were primarily investigated in this research. A total of 240 Pekin ducks were used, and they were reared until slaughter age (42 d) in three different stocking density groups (three, five and seven ducklings m ${}^{-2}$ ). To compare the slaughter performance of the ducklings' live weight, carcass weight, carcass yield, thigh and breast meat weight and yield, and edible giblet weight (heart, liver and gizzard) were investigated. The meat quality was compared between the treatment groups based on dry matter ratio, cooking loss, water-holding capacity, pH values, and colour parameters ( L , a , b , c , h and ΔE values). Carcass weight, carcass yield, thigh and breast meat weight were found to decrease in parallel to the increasing stocking density, resulting in a reduction in thigh and breast meat weights and ratios ( P<0.05 ). Increasing the stocking density decreased the heart weight and positively improved the liver and gizzard ratio ( P<0.05 ). However, it did not affect the meat quality parameters investigated in this research ( P<0.05 ). The breast meat of the ducks reared under higher stocking density had higher L , h and ΔE values, lower a value ( P<0.05 ), and similar b and c values ( P<0.05 ). Evaluating the overall research findings, it was concluded that increased stocking density when rearing ducks negatively affects the slaughter performance, affecting only breast meat colour and weight of thigh meat with skin in investigated meat quality parameters.

## 1 Introduction

Ducks (*Anas platyrhynchos domestica*) are more resistant to cold, hot and humid environments than most other poultry species, especially chicken, making them more durable and easier to grow for both farmers and commercial producers (Wright, 2008; Holderread, 2011; HTE Books, 2016; Ekarius, 2007). The demand for duck meat has visibly increased in the last decade. In 2010, 2.1 billion ducks were reared, and 4.0 $\times 10^{6}$ t of duck meat was produced (FAO, 2010) where from 2010 to 2021 production is reported to be increasing 3 % yearly (Yahoo Finance, 2022). Pekin duck is the primary duck meat source for the European Union (EU). In the EU, especially in France, Poland, Hungary and Germany, duck production is undertaken intensively and in some other countries within the EU extensively. Through years of selection and breeding, several hybrid lines have been formed with lower fat deposits, higher weight gain, and better carcass yield (CY) and field performance (Wencek et al., 2012; Grimaud Freres Selection S.A.S, 2016). The slaughter weight of earlier ducks was 2.00–2.50 kg at 7 weeks using two types of feed (Dogan, 1987; Testik et al., 1987). This value first increased to 3195 g (Leeson et al., 1980), then to 3342 g (Knizetova et al., 1991) and finally to 3750 g in more recent years (Holderread, 2011).

CY and the weight of edible components, such as liver, heart, and gizzard, are important parameters of meat and slaughter performance in duck production. Duck liver is a very important source of income for producers and has a big market in Hungary and is mostly acquired from the hybrids of Pekin and Muscovy ducks (Holderread, 2011) around Europe.

The most common production period is 7–9 weeks, during which mostly two types of feed are used as the starter feed for the first 2 weeks and the grower–finisher feed for the last 5 weeks. When the ducks are reared for 9 weeks for meat production, the starter feed is used for the first 2 weeks, the grower feed for the next 5–6 weeks, and finisher diets are used in the last 1–2 weeks (Knizetova et al., 1991).

As previously reported, Pekin ducks grow better in free-range systems with access to swimmable water sources. With the growing poultry sector and industrialisation in production, environmentally controlled rearing houses have been commonly used worldwide to produce hybrid Pekin ducks with higher stocking density (SD), and recently, it was shown that ducks also grow well in industrial systems, such as floor rearing using different litter materials in commercial houses (Reiter et al., 1997; Chen et al., 2015; Jones et al., 2010).

A basic standard for duck production published by the EU (Council of Europe, 1999) recommended producing ducks with an SD of 23.50 kg m ${}^{-2}$ for a mean live weight (MLW) of 3.35 kg (DEFRA, 2007). However, there is still a very large information gap about this topic. In the light of the studies mentioned, this experiment was conducted to understand the effects of SD on the slaughter performance and meat quality of Pekin ducks and obtain more detailed data to contribute to science and further research.

## 2 Material and method

A total of 240 mixed-sex, day-old Grimaud Star 53 Pekin ducklings were obtained from a private duck meat production company in Bolu, Türkiye, for use in the experiment. The ducklings were reared in a research and development (R&D) house of a private duck growing facility with permission of the owner and the production company.

There were 12 trial pens in the fully automated, environmentally controlled R&D house heated by four pieces of 3000 W electric oil radiator heaters (Flavel RI 3000M, Türkiye) and ventilated by a total of three tunnel fans, including two minimum ventilation fans with a flow of 1100 m ${}^{3}$ h ${}^{-1}$ (Bahcivan BPP 30, Türkiye) and one cooling fan with a flow of 4500 m ${}^{3}$ h ${}^{-1}$ (Bahcivan BSM 400, Türkiye), specifically chosen and mounted for the volume and insulation of the R&D house to achieve minimum ventilation and cooling when needed.

The day-old ducklings were weighed upon arrival at the farm's R&D house. Then, they were placed in pens having an area of 4 m ${}^{2}$ according to the trial plan, achieving three, five and seven ducklings m ${}^{-2}$ SD randomly. Pan feeders with a capacity of 10 kg feed and duck and broiler nipple drinkers (three nipples per 50 cm of pipeline) connected to individual water tanks pre-partitioned for easy measurement of water consumption were used in every single trial pen. The nutritive value of the feed was given in Table 1. The pens were 2×2 m in dimensions. The lighting system of the house consisted of 12 LED bulbs standing on each trial pen to achieve 75 lx maximum illumination at the beginning of the rearing period and dimmed after the first week to 30 lx and maintained until the end of the rearing period. For the first 3 d of life, the lights were kept on, then darkening began with 30 min d ${}^{-1}$ , and the dark period was increased by 30 min every day until it reached 10 h on the 23rd day and maintained to the end of the rearing period. The starting rearing house temperature of the growing period was 32 ${}^{\circ }$ C, which was decreased by 0.5 ${}^{\circ }$ C every day until reaching 20 ${}^{\circ }$ C on day 25 and maintained at 20 ${}^{\circ }$ C until the end of the rearing period. The R&D house automated control system was specifically built for the R&D house to keep the intra-climate stable and automatically control heaters, ventilation, cooling and lighting systems throughout the production term. The measurements were checked every 4 h, and changes were made when needed.

**Table 1:** Nutritive value of the feed used in the trial.

	Starter	Grower
	0–14 d	15 d–slaughter
Metabolic energy, kcal kg ${}^{-1}$	2900.00	3100.00
Crude protein, %	20.00	17.20
Crude cellulose, %	4.00	4.05
Crude fat, %	4.13	5.81
Crude ash, %	6.33	6.33
Lysine, %	1.00	0.80
Methionine, %	0.55	0.40
Calcium, %	1.00	0.90
Phosphorus, %	0.72	0.65
Sodium, %	0.16	0.17
Vitamin A, IU	12 000.00	12 000.00
Vitamin D3, IU	5000.00	5000.00
Manganese, mg kg ${}^{-1}$	120.00	120.00
Zinc, mg kg ${}^{-1}$	110.00	110.00
Copper, mg kg ${}^{-1}$	16.00	16.00
Iodine, mg kg ${}^{-1}$	1.50	1.50
Selenium, mg kg ${}^{-1}$	0.30	0.30

At the end of the rearing period, eight male ducks from each treatment group, two from each rearing pen, were randomly chosen and transferred to the slaughterhouse of the company. The ducks were slaughtered in the company's integrated facility by hand on conveyors hanging stainless-steel holders as described by Farouk et al. (2014) according to the Islamic halal standards and EU legislation. After slaughter, the necks of the birds were removed and not included in any further measurement. After letting the blood drain, the hot carcass weights (CWs) were determined. After 15 min, the weights of thigh meat with skin (STW), thigh meat without skin (SlTW), breast meat with skin (SBW), breast meat without skin (SlBW) and edible giblet (heart weight (HW), liver weight (LW) and gizzard weight (GW)) were measured by a normal ( ± 1 mg) scale (TEM TNT 015D, Türkiye). Using these data, CY and the yields of edible giblets (heart yield (HY), liver yield (LY), and gizzard yield (GY)) were calculated.

**Table 2:** The effects of stocking density on the slaughter performance of Pekin ducks (mean ± SEM).

	Stocking density (SD) ducks m ${}^{-2}$			
	3	5	7	P value
Total live weight, kg m ${}^{-2}$	10.71 ± 0.26 ${}^{b}$	14.62 ± 0.43 ${}^{b}$	17.43 ± 1.16 ${}^{a}$	0.000
Slaughter weight, g	3578 ± 105 ${}^{a}$	2924 ± 73 ${}^{b}$	2491 ± 168 ${}^{b}$	0.000
Carcass weight, g	1987 ± 66 ${}^{a}$	1562 ± 50 ${}^{b}$	1172 ± 35 ${}^{c}$	0.000
Carcass yield, %	55.51 ± 0.81 ${}^{a}$	53.38 ± 0.68 ${}^{a}$	47.90 ± 1.97 ${}^{b}$	0.002
Fragment weight, g				
Thigh (with skin)	546 ± 24 ${}^{a}$	465 ± 14 ${}^{b}$	342 ± 14 ${}^{c}$	0.000
Breast (with skin)	504 ± 18 ${}^{a}$	357 ± 22 ${}^{b}$	200 ± 14 ${}^{c}$	0.000
Thigh (without skin)	409 ± 17 ${}^{a}$	354 ± 9 ${}^{b}$	274 ± 7 ${}^{c}$	0.000
Breast (without skin)	373 ± 18 ${}^{a}$	238 ± 15 ${}^{b}$	123 ± 6 ${}^{c}$	0.000
Thigh skin	137 ± 13 ${}^{a}$	111 ± 11 ${}^{b}$	68 ± 8 ${}^{b}$	0.001
Breast skin	130 ± 8 ${}^{a}$	119 ± 9 ${}^{b}$	76 ± 12 ${}^{b}$	0.002
Fragment yield, carcass %				
Thigh (with skin)	32.66 ± 1.40 ${}^{a}$	27.78 ± 0.83 ${}^{b}$	20.44 ± 0.82 ${}^{c}$	0.000
Breast (with skin)	24.49 ± 1.01 ${}^{a}$	21.16 ± 0.55 ${}^{b}$	16.39 ± 0.42 ${}^{c}$	0.000
Thigh (without skin)	30.11 ± 1.07 ${}^{a}$	21.33 ± 1.31 ${}^{b}$	11.93 ± 0.82 ${}^{c}$	0.000
Breast (without skin)	22.31 ± 1.09 ${}^{a}$	14.23 ± 0.89 ${}^{a}$	7.38 ± 0.38 ${}^{c}$	0.000
Thigh skin	6.92 ± 0.65	7.09 ± 0.66	5.71 ± 0.54	0.241
Breast skin	6.60 ± 0.39	7.54 ± 0.38	6.56 ± 1.02	0.527
Edible giblet weight, g				
Liver	76 ± 6	78 ± 3	84 ± 4	0.460
Heart	17 ± 1 ${}^{a}$	14 ± 1 ${}^{b}$	11 ± 1 ${}^{c}$	0.000
Gizzard	101 ± 3 ${}^{a}$	83 ± 3 ${}^{b}$	76 ± 4 ${}^{b}$	0.000
Edible giblet yield, weight %				
Liver	2.11 ± 0.12 ${}^{c}$	2.68 ± 0.09 ${}^{b}$	3.43 ± 0.22 ${}^{a}$	0.000
Heart	0.48 ± 0.02	0.87 ± 0.02	0.44 ± 0.02	0.504
Gizzard	2.82 ± 0.08	2.84 ± 0.14	3.09 ± 0.20	0.366
Edible giblet yield, carcass %				
Liver	3.81 ± 0.24 ${}^{c}$	5.04 ± 0.20 ${}^{b}$	7.14 ± 0.30 ${}^{a}$	0.000
Heart	0.86 ± 0.05	0.87 ± 0.02	0.94 ± 0.05	0.404
Gizzard	5.07 ± 0.10 ${}^{\mathrm{cb}}$	5.34 ± 0.28 ${}^{b}$	6.46 ± 0.34 ${}^{a}$	0.004

${}^{\mathrm{abc}}$ Different superscript letters on the same line indicate statistical significance ( P<0.05 ).

Dry matter (DM), pH, water-holding capacity (WHC), cooking loss (CL) and colour analyses were also performed to investigate the effects of different treatments on meat quality. To obtain DM data, the DM plates were dried at 105 ${}^{\circ }$ C, and then the tares of these plates were measured. A laboratory scale ( ± 0.01 g) was used (Radwag AS220R2, Poland) to weigh 5 g samples. These samples were then placed on the DM plates and dried at 105 ${}^{\circ }$ C until a stable weight was achieved and reweighed to obtain the water content of the meat before the procedure. The process was applied as explained by Association of Official Analytical Chemists (1990) and Jensen et al. (2004).

The pH level of the thigh and breast meat was measured by an automatic pH meter (WTW 3110, Germany) using a glass pH probe (WTW Sentix 31, Germany). Before taking the measurements, the device was calibrated by ready-to-use pH 4 and pH 7 solutions. The measurements were separately undertaken from the same locations of the breast and thigh 4 h after slaughter and cooling at + 4 ${}^{\circ }$ C for 24 h.

The WHC values were obtained by the filter paper press method as recommended by Grau et al. (1953) from the same parts of the thigh and breast meat as the DM samples were taken. A total of 300 mg of samples was weighed, placed between filter paper and millimetre paper, numbered individually, and pressed between two special plexiglass plates for 3 min. The measurements were taken twice: after 24 and 48 h of slaughter. The samples between the two pieces of paper were taken out of the press and stapled in the corner of the paper. A tripod was set up inversely on the laboratory table, and a digital SLR camera (Canon T1i, Japan) with a lens of 18–55 f was attached to this tripod to obtain macro photos from the surface of the table under standard lighting conditions using a light box. The prepared samples were placed under this platform and photographed. These photos were transferred to the computer, and JI (JImage Imaging Software – National Institutes of Health 2022, USA) area calculation software was used to determine the area of meat and the area of water spread. The area of spread meat was extracted from the area of spread water to obtain the free water area. From the measurements performed, the WHC values were calculated as a percentage by dividing the free water area by the total area.

**Table 3:** The effects of stocking density on the meat quality of Pekin ducks (mean ± SEM).

	Stocking density (SD) ducks m ${}^{-2}$			
	3	5	7	P value
pH				
Thigh, slaughter	6.60 ± 0.12	6.61 ± 0.10	6.41 ± 0.10	0.297
Thigh, 24 h	6.65 ± 0.06	6.66 ± 0.05	6.43 ± 0.11	0.085
Breast, slaughter	5.91 ± 0.06	5.92 ± 0.07	6.01 ± 0.07	0.528
Breast, 24 h	5.85 ± 0.02	5.99 ± 0.13	5.79 ± 0.06	0.207
Water-holding capacity, %				
Thigh, Day 1	0.60 ± 0.05	0.62 ± 0.02	0.62 ± 0.04	0.936
Thigh, Day 2	0.57 ± 0.03	0.61 ± 0.07	0.65 ± 0.09	0.673
Breast, Day 1	0.75 ± 0.02	0.76 ± 0.02	0.83 ± 0.03	0.143
Breast, Day 2	0.71 ± 0.02	0.73 ± 0.02	0.82 ± 0.05	0.124
Dry matter ratio, %				
Thigh	23.55 ± 0.70	24.17 ± 0.88	23.33 ± 0.55	0.691
Breast	23.20 ± 0.34	23.24 ± 0.37	22.23 ± 0.50	0.178
Cooking loss, %				
Thigh	16.90 ± 0.93	15.80 ± 1.19	15.55 ± 1.19	0.603
Breast	21.27 ± 1.44	19.42 ± 1.21	19.27 ± 0.92	0.441

${}^{\mathrm{abc}}$ The different superscript letters on the same line indicate statistical significance ( P<0.05 ).

To obtain the CL data, a laboratory ( ± 1 mg) scale (Radwag AS220R2, Poland) was used, and the process was carried out as described by Honikel (1998). A total of 50 g of samples was taken from the thigh and breast meats of the ducks (approximately 3 cm wide, 3 cm high and 5 cm long), weighed by the scale, and placed in a plastic heat-resistant sealed bag. The bags were individually numbered by water-resistant pen. The bags were immersed in a boiling water bath and kept there until the centre of the meat reached 75 ${}^{\circ }$ C. At this point, the samples were taken out of the boiling water and immersed in cold water with ice until they reached room temperature. Then, the meat was removed from the bag and weighed. The weight loss between the first and last measurements was calculated as the percentage of the CL value.

The colour measurements were taken from the thigh meat with skin (ST), breast meat with skin (BS), thigh without skin (S1T) and breast without skin (SlB) using a portable digital colorimeter (PCE_CSM5, PCE Instruments, USA). The colorimeter was precalibrated by a white and black plate provided. The colorimeter was also double-checked by another colorimeter (Minolta CR-400, Osaka, JAPAN) by measuring 10 samples in parallel and found to deliver the same measurement values. The L , a , b and hue ( h ) values were obtained from the automatic colorimeter as colour parameters, and these data were used to calculate the ΔE ( $L^{2}+a^{2}+b^{2}$ ) and chroma ( C=√ ( $a^{2}+b^{2}$ )) values. The colour parameters were tested by the CIELab system with L value meaning dark to light (0–100), a value meaning green to red ( - 60 to + 60), and b value blue to yellow ( - 60 to + 60).

All meat quality evaluations were performed as described in the meat quality evaluation book of Anadolu University (Kivanç, 2010) and Nollet et al. (2009).

The trial was set up according to a random-parcel plan. After gathering all the data and ensuring data were homogeneous by applying the homogeneity tests (skewness and kurtosis analysis followed by the Shapiro–Wilk test), statistical analyses were performed on a computer using the ANOVA method and the post hoc Tukey test using IBM SPSS 22 software program (SPSS, 2013, USA). The findings were obtained as means ± standard error of the means (SEM).

**Table 4:** The effects of stocking density on the meat colour of Pekin ducks (mean ± SEM).

	Stocking density (SD) ducks m ${}^{-2}$			
	3	5	7	P value
L				
Thigh (with skin)	72.77 ± 0.75	70.59 ± 1.13	70.69 ± 1.77	0.459
Breast (with skin)	72.74 ± 4.29	73.46 ± 3.89	79.08 ± 0.76	0.343
Thigh (without skin)	50.10 ± 5.37	46.42 ± 0.48	47.47 ± 1.71	0.689
Breast (without skin)	49.24 ± 2.16 ${}^{b}$	51.69 ± 1.88 ${}^{\mathrm{ab}}$	56.22 ± 1.57 ${}^{a}$	0.047
$a^{*}$ value				
Thigh (with skin)	4.99 ± 0.62 ${}^{a}$	3.18 ± 0.28 ${}^{b}$	3.29 ± 0.46 ${}^{b}$	0.022
Breast (with skin)	6.95 ± 1.40	5.49 ± 0.59	4.50 ± 0.60	0.183
Thigh (without skin)	10.19 ± 1.47	11.62 ± 0.67	11.25 ± 0.61	0.563
Breast (without skin)	11.13 ± 0.65	10.44 ± 0.38	9.29 ± 0.50	0.059
$b^{*}$ value				
Thigh (with skin)	6.77 ± 0.66	5.08 ± 1.10	5.62 ± 1.12	0.508
Breast (with skin)	11.30 ± 1.24	11.73 ± 1.62	13.36 ± 0.59	0.471
Thigh (without skin)	5.22 ± 1.78	5.66 ± 0.64	3.00 ± 1.35	0.277
Breast (without skin)	3.64 ± 0.65	4.21 ± 0.54	4.91 ± 0.31	0.241
$c^{*}$ value				
Thigh (with skin)	8.55 ± 0.65	6.14 ± 1.02	6.71 ± 1.05	0.207
Breast (with skin)	13.83 ± 2.59	13.41 ± 1.12	14.13 ± 0.74	0.862
Thigh (without skin)	12.16 ± 1.01	12.96 ± 0.84	12.06 ± 0.90	0.739
Breast (without skin)	11.66 ± 0.78	11.33 ± 0.45	10.57 ± 0.38	0.365
$h^{*}$ value				
Thigh (with skin)	53.82 ± 4.13	52.74 ± 5.53	55.44 ± 5.95	0.935
Breast (with skin)	59.27 ± 6.71	61.68 ± 6.91	71.89 ± 1.51	0.252
Thigh (without skin)	29.32 ± 9.71	25.52 ± 1.78	19.44 ± 4.36	0.505
Breast (without skin)	15.43 ± 2.70 ${}^{b}$	21.72 ± 2.43 ${}^{\mathrm{ab}}$	28.25 ± 2.44 ${}^{a}$	0.007
Δ value				
Thigh (with skin)	73.29 ± 0.75	70.90 ± 1.19	71.06 ± 1.81	0.459
Breast (with skin)	74.13 ± 4.14	74.70 ± 3.97	80.36 ± 0.70	0.343
Thigh (without skin)	51.71 ± 5.21	48.24 ± 0.62	49.01 ± 1.80	0.699
Breast (without skin)	48.24 ± 0.62 ${}^{b}$	52.95 ± 1.85 ${}^{\mathrm{ab}}$	57.23 ± 1.50 ${}^{a}$	0.046

${}^{\mathrm{abc}}$ The different superscript letters on the same line indicate statistical significance ( P<0.05 ).

## 3 Results and discussion

The focus of this research was to investigate the effects of SD on slaughter performance, carcass quality, and some meat quality parameters in hybrid Pekin ducks. To investigate the slaughter performance, the main criteria were TLW (total live weight m ${}^{-2}$ ), SW (slaughter weight), CW and CY. All slaughter performance parameters were adversely affected by increasing the SD and the worst values were observed in the highest SD of seven ducks per square metre as shown in Table 2. These data results are in line with previous research, reporting that the ducks reared reached 3.350 kg in around 48 d (Jones et al., 2010) and 3.518 kg in 49 d (Steczny et al., 2017), like the treatment groups of the current research (2.491, 2.924 and 3.580 kg in 42 d). The weights of the ducks being lower in some of the treatment groups of the current study are considered to be due to the shorter duration of the rearing period in the trial. If the rearing period had been 48 d, all treatment groups would have reached and may have even exceeded the weights reported in the literature.

The weights of the ducks' body components investigated, which were thigh weigh without skin (SlT), breast meat without skin (SlB), ST, BS, SlTW and SlBW, were found to gradually decrease in parallel to the increasing SD (Table 2). The percentages of breast meat and thigh meat in carcass were 14.23 % and 21.33 %, respectively, which were higher than found in the study by Xie et al. (2014) reporting the breast and thigh meat percentages as 13.4 % and 13.6 %, respectively. It is first thought that these differences might have been due to the total carcass weight being lower in our study since we did not include the neck in measurements. However, even when we adjusted the values, our values were higher, which suggests that the rearing conditions and management were better than the other research undertaken, and the race and lines of birds used in the current trial were fine.

The mean CY was found to be 52.26 % in the experiment, which was lower than the findings of previous experiments, e.g. 72.1 % (İşgüzar, 2006), because we did not include the feet, neck, and head in the total carcass weight. As reported by some researchers, the head of the ducks constitutes around 5.20 % of LW and 7.23 % of carcass, the necks of the ducks around 7.30 % of carcass, and the feet around 3.47 % of carcass. This means that in the current study, approximately 18.00 % of carcass weight was not evaluated. This was to eliminate the fluctuation of the total carcass measurements. The total CY in the current trial was around 71.00 %, which is consistent with previous reports of as İşgüzar (2006) (72.10 %) and Steczny et al. (2017) (70.25 %).

When the weights of edible giblets were analysed in relation to SD, a similar situation emerged. The heart and gizzard weights showed a tendency to decrease with the increasing SD ( P<0.05 ), and the lowest values were obtained from the SD of seven ducks per square metre (Table 2). In contrast, the liver weights were observed to increase with the increasing SD, but the differences were not significant ( P<0.05 ).

When the yield data of the edible giblets were analysed based on the percentage to total live weight, liver yield was found to increase ( P<0.05 ) with the increasing SD, reaching the highest in the SD of seven ducks per square metre. However, the heart and gizzard yields were not affected by SD ( P<0.05 ) (Table 2).

The analysis of edible giblet yield data in relation to the whole carcass revealed that the liver and gizzard yields increased in parallel to the increase in SD ( P<0.05 ), but the heart yield was found to be unaffected by SD in the experiment in percentage to whole carcass ( P<0.05 ). These data about edible giblets were in line with other research's findings where percentages of giblets were reported as 0.86 % heart, 1.13 % liver and 2.03 % gizzard by Staczny et al. (2017) and 0.88 % heart, 2.69 % liver and 6.41 % gizzard by İşgüzar (2006).

None of the investigated meat quality parameters (pH, WHC, dry matter ratio, and cooking loss) were affected by SD ( P<0.05 ) (Table 3). The pH values obtained from the research were 6.41–6.61 for thigh meat and 5.91–6.01 for breast meat, which agrees with the literature (Chen et al., 2015 (6.04 and 6.09); Ahaotu et al., 2015 (5.96–6.25); Michalczuk et al., 2016 (5.90)).

Concerning meat colour, the L , h and Δ values increased only in skinless breast meat in parallel to the increasing SD ( P<0.05 ). The colour values of the thigh meat with skin were higher in the SD of three ducks per square metre ( P<0.05 ), meaning that as the SD was reduced, the colour of the thigh meat with skin became more reddish. Other parameters were not affected by the different SDs applied in the research ( P<0.05 ) (Table 4). Therefore, it can be stated that increasing the SD resulted in the skinless breast meat to become lighter in colour and increased the h and Δ values.

## 4 Conclusions

When the results of the research were evaluated in general, it was seen that the increase in the SD affected the slaughter performance criteria negatively, but in contrast most criteria related to meat quality were not affected. As a criterion for meat quality, the effect of SD on the colour of meat was only observed in the skinless breast meat having a lighter colour and the thigh meat with skin being reddish in the lower SD groups. Considering that these parameters influence consumer demand in the market, more attention must be paid to determine the optimal SD to increase not only field and slaughter performance, but also meat quality through more detailed and large-scale studies that also take into consideration economic parameters.

## Data Availability

All raw data can be provided by the corresponding authors upon request.

## References

[bib1.bib1] Ahaotu EO, Agbasu CA (2015). Evaluation of the stocking rate on growth performance, carcass traits and meat quality of male pekin ducks. Sci J Biol Sci.

[bib1.bib2] Association of Official Analytical Chemists (1990). Official Methods of Analysis of Association of Official Chemists.

[bib1.bib3] Chen Y, Aorigele C, Yan F, Li Y, Cheng P, Qi Z (2015). Effect of production system on welfare traits, growth performance and meat quality of ducks. South Afr J An Sci.

[bib1.bib4] Council of Europe (1999). Council of Europe, Standing Committee of the European convention for the protection of animals kept for farming purposes.
Recommendations concerning Muscovy Ducks (Cairina moschata) and hybrids of Muscovy and Domestic ducks (Anas platyrhynchos), adopted by the Standing Committee on 22 June 1999.

[bib1.bib5] (2007). Codes of Recommendation for the Welfare of Livestock: Ducks.

[bib1.bib6] Dogan K (1987). Feeding and Meat Producing of White Pekin Ducks. Feed Ind J.

[bib1.bib7] Ekarius C (2007). Storey's Illustrated Guide to Poultry Breeds.

[bib1.bib8] FAO (2010). Duck livestock data.

[bib1.bib9] Farouk MM, Mazeedi HMA, Sabow AB, Bekhit AED, Adeyemi KD, Sazili AQ, Ghani A (2014). Halal and Kosher methods and meat quality: A review. Meat Sci.

[bib1.bib10] Grau R, Hamm R, Baumann A (1953). Uber das Wasserbindungsvermögen des totenSäugetiermuskels. I Biochem J.

[bib1.bib11] Grimaud Freres Selection SAS (2016). Rearing Guide Roasting Pekin Ducks.

[bib1.bib12] Holderread D (2011). Storey's Guide to Raising Ducks: Breeds, Care, Health.

[bib1.bib13] Honikel KO (1998). Reference methods for the assessment of physical characteristics of meat. Meat Sci.

[bib1.bib14] HTE Books (2016). How to Raise Strong & Healthy Ducks.

[bib1.bib15] İşgüzar E (2006). Isparta Yöresi Karışık Yerli Ördek Genotipleri ve Pekin Ördeklerinde Yerleşim Sıklığının Büyüme ve Karkas Özelliklerine Etkileri. Süleyman Demirel Üniversitesi Fen Bilimleri Enstitüsü Dergisi.

[bib1.bib16] Jensen WK, Devine C, Dikeman M (2004). Encyclopedia of Meat Science.

[bib1.bib17] Jones TA, Dawkins MS (2010). Environment and Management Factors Affecting Pekin Duck Production and Welfare on Commercial Farms in the UK. British Poult Sci.

[bib1.bib18] Kivanç M (2010). Et ve Et Ürünlerinin Kalite Kontrolü, Vol 224.

[bib1.bib19] Knizetova H, Hyanek B, Knize B, Prochaskova H (1991). Analyses of Growth Curves of Fowl (Ducks). British Poult Sci.

[bib1.bib20] Leeson S, Summers JD (1980). Production and Carcass Characteristics of The Broiler Chicken. Poult Sci.

[bib1.bib21] Michalczuk M, Damaziak K, Pietrzak D, Marzec A, Chmiel M, Adamczak L, Florowski T (2016). Influence of housing system on selected quality characteristics of duck meat. Chapter 1. Pekin duck. Annals of Warsaw University of Life Sciences. SGGW Animal Science.

[bib1.bib22] Nollet LML, Toldra F (2009). Handbook of muscle food analysis.

[bib1.bib23] Reiter K, Zernig F, Bessei W (1997). Effect of Water Bath and Free Range on Behaviour and Feathering in Pekin, Muscovy and Mulard Duck.

[bib1.bib24] SPSS (2013). IBM Corp, Released 2013, IBM SPSS Statistics for Windows, Version 22.0.

[bib1.bib25] Steczny K, Kokoszynski D, Bernacki Z, Wasilewski R, Saleh M (2017). Growth performance, body measurements, carcass composition and some internal organ characteristics in young Pekin ducks. South Afr J An Sci.

[bib1.bib26] Testik A, Pekel E, Sarica M (1987). A Study on Growing Performance of Pekin Duck.

[bib1.bib27] Wencek E, Kałużna I, Koźlecka M, Prokopiak H, Adamski M (2012). Results of poultry performance recording in 2011.

[bib1.bib28] Wright L (2008). Choosing and Keeping Ducks and Geese.

[bib1.bib29] Xie M, Jiang Y, Tang J, Wen ZG, Huang W, Hou SS (2014). Effects of stocking density on growth performance, carcass traits and foot pad lesions of White Pekin ducks. Poult Sci.

[bib1.bib30] (2022). Duck Meat Market by Product and Geography – Forecast and Analysis 2021–2025 [Internet].

